# Future of Orthodontics—A Systematic Review and Meta-Analysis on the Emerging Trends in This Field

**DOI:** 10.3390/jcm12020532

**Published:** 2023-01-09

**Authors:** Mohammad Khursheed Alam, Huda Abutayyem, Bushra Kanwal, Maher A. L. Shayeb

**Affiliations:** 1 Orthodontic Division, Preventive Dentistry Department, College of Dentistry, Jouf University, Sakaka 72345, Saudi Arabia; 2 Department of Dental Research Cell, Saveetha Institute of Medical and Technical Sciences, Saveetha Dental College and Hospitals, Chennai 72345, India; 3 Department of Public Health, Faculty of Allied Health Sciences, Daffodil lnternational University, Dhaka 1216, Bangladesh; 4 Center of Medical and Bio-Allied Health Sciences Research, Department of Clinical Sciences, College of Dentistry, Ajman University, Ajman P.O. Box 346, United Arab Emirates; 5 Practicing in Dental Clinic, Al Baha 65511, Saudi Arabia

**Keywords:** 3D printing, aligners, artificial intelligence, future trends, orthodontics, teleorthodontics

## Abstract

Technology is rapidly evolving in the modern world, and the accompanying developments due to its influence are shaping each and every aspect of our life, with the field of orthodontics being no exception. This systematic review and meta-analysis aimed to examine such trends in orthodontics and hypothesize which ones would emerge and continue in the near future. After a thorough search of online journals using keywords such as “3D printing,” “Aligners,” “Artificial intelligence,” “Future trends,” “Orthodontics,” and “Teleorthodontics” across databases of PubMed-MEDLINE, Web of Science, Cochrane, and Scopus, a total of 634 papers were initially recovered. Technological advancements in 3D printing, Computer-aided design and Computer-aided manufacturing (CAD/CAM), biopolymers and Teleorthodontics were the most important categories of development seen across the 17 studies that we selected for our review. All the investigations selected for this systematic review depicted aspects of orthodontics that were influenced by rapid technological changes and could potentially become mainstream in the coming times. However, caution was sought to be observed in the usage/adoption of some of these trends, with social media usage amongst both patients as well as orthodontists being a prime example of this.

## 1. Introduction

Dental problems such crooked teeth, misaligned jaws, and abnormal bite patterns are the focus of the science of orthodontics, which also focuses on their diagnosis, prevention, and treatment [1,2]. Jaw and tooth misalignments are currently a very common issue. According to the American Association of Orthodontics (AAO), 50% of people have malocclusions severe enough to require orthodontic care. This number drops to less than 10% when implanting orthodontics that are medically necessary, according to the same AAO statement [3,4]. There is not enough credible scientific evidence to support the benefits of orthodontic treatment for health. Numerous researchers are attempting to resolve this significant orthodontic issue by developing new materials and methods [5]. A few months to a few years may pass during the course of treatment, and braces and other appliances will be used to gradually realign the teeth and jaws. Jaw surgery might be required in situations with severe malocclusion. Children’s bones are more pliable than adults’, thus starting treatment before the child reaches maturity may make things easier and reduce challenges [6,7].

With the development of advanced manufacturing technologies, research, and design, as well as the rising popularity of three-dimensional (3D) imaging modalities, the implementation of this rapidly evolving technological aspect of society has advanced noticeably over the past ten years. It has now permeated every technological area, including industrial fields, manufacturing processes, military applications, medical fields, and research where conventional methods are employed. Mobile applications (apps), for instance, are expected to play a significant part in the management of modern pleasant and appealing therapies, where patient compliance is crucial. In addition to the usual verbal encouragement given by orthodontists to young patients undergoing orthodontic treatment, patients’ social networks such as Instagram are already playing an increasingly crucial role in daily life [8]. It is evident that both doctors and patients stand to benefit greatly from this technology given the emergence of apps linked to orthodontics and the speedy development of artificial intelligence. More advanced AI technologies have lately become available for orthodontic applications. With the help of technologies such as three-dimensional convolutional neural networks (3D CNN), a huge potential for automated 3D cephalometric evaluation straight from cone-beam computed tomography (CBCT) or face growth forecasts exists [9].

Another area where technology has immensely aided medical practitioners is in imaging and diagnosis. Imaging is a crucial and important part of diagnostic and planning in orthodontics. When necessary, intraoral radiographs have been utilized as a backup for panoramic and cephalometric radiographs, which have historically been employed largely for initial diagnostic and therapeutic follow-up evaluation. The CBCTs have proven to be of great assistance to patients with complex oral and maxillofacial disorders [10]. CBCT allows for a more thorough understanding of the patient’s anatomy, and the data from these images can be integrated with images and 3D surface models to produce dynamic, patient-specific anatomical reconstructions and the potential for 3D treatment planning. It is possible to develop a number of tools using algorithms to transform the raw data from these images into sizable data sets and possibly apply artificial intelligence to find anatomical differences and/or diseases [11] because of the enormous variety of structures that are visible on these images. Many recent studies are focusing on mining the anatomical structure data based on predefined imaging features such as signal-to-noise ratio, windowing, and levelling to enable the use of artificial intelligence to help detect subtle changes in anatomy and any incipient lesions which may not have been picked up by humans. The method has limited application and is not yet ready for use in the craniofacial area, typically concentrating on a few disorders. Due to the complexity of the structures in the craniofacial region and the high prevalence of incidental findings on CBCT images, an expert in radiologic interpretation of the oral and maxillofacial complex should assess the images in order to detect the presence of abnormal conditions and/or anatomical variations. This can be facilitated very well by artificial intelligence [12].

Additionally, the distinctive features of the COVID-19 pandemic outbreak have illustrated the importance of health issues for the entire community [13]. Due to social distance, only essential services were kept open during the spring lockout in 2020. The COVID-19 pandemic had a significant impact on oral dental professionals due to the widespread closure of dental clinics [14,15]. The pandemic’s effects have persisted since orthodontic therapy is a drawn-out process that requires repeated visits [16]. Due to this, a large number of orthodontic patients who were already receiving treatment skipped their monthly sessions [17].

Hence, our primary objective with respect to conducting this systematic review and meta-analysis was to examine articles from the orthodontic literature that described current practices that are expected to become more common in the near future as well as future trends in the industry.

## 2. Materials and Methods

This study was registered to the International Prospective Register of Systematic Reviews (PROSPERO), registration number: CRD42022378377.

### 2.1. Protocol Employed

This systematic review was performed as per the Preferred Reporting Items for Systematic Review and Meta-analysis (PRISMA) strategy and rules from the Cochrane group and the book Orderly reviews in Health care: Meta examination [18].

### 2.2. Review Hypotheses

Through this systematic review, our primary objective was to review studies that were published in the orthodontic literature and that discussed the future trends in this field and shed light upon existing practices as well, which are supposed to become mainstream in the coming times.

### 2.3. Study Selection

**Figure 1:**
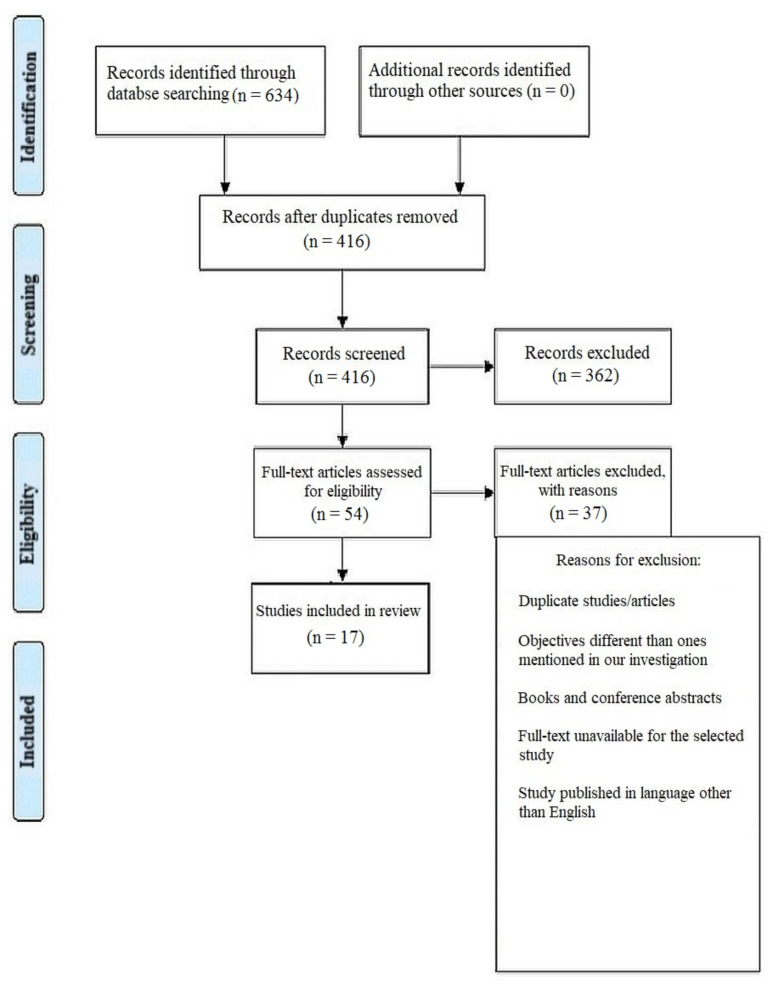
Representation of selection of articles through PRISMA framework.

There was a total of 634 documents discovered after extensive search on the online journals and 416 of the papers were selected initially. Following that, 362 similar/duplicate articles were eliminated, which resultantly made 54 separate papers available at first. The abstracts and titles of submissions were then reviewed, and a further 37 papers were eliminated. Finally, 17 documents that met the requisite inclusion and exclusion criteria were chosen, which primarily included in-vitro studies, literature reviews and comparative assessments (Figure 1).

### 2.4. Inclusion Criterion

Articles that contained relevant data for our review objectives were selected for full-text screening. Studies that reported clinical trials, in-vitro studies, systematic/literature reviews containing substantial sample volume and detailed case reports were considered for inclusion in our review. We also monitored studies that possessed higher methodological quality.

### 2.5. Exclusion Criteria

The following were excluded from the scope of our systematic review: incomplete data, seminar presentations, scholarly articles, placebo-controlled studies, and opinion articles.

Since the literature available on this topic was quite scant in volume, we did not limit our search in terms of the time period when the studies were published, i.e., we took into account all the papers that were published with context to our topic (where the number of papers itself was found to be quite sparse in number). In addition, literature reviews and cases published in languages other than English were excluded.

We refrained from selecting any randomized control trials for our study, since we believe ours is a speculative investigation, analyzing the upcoming advances in the field of orthodontics that have the potential to be implemented in future practice, and as such it would be too early to analyze studies that implement the use of these technologies/advancements in human subjects. Moreover, studies involving the assessment of these trends on people were found to be few and far between with poor methodological value, hence their exclusion.

### 2.6. Search Strategy

Using relevant keywords, reference searches, and citation searches, the databases PubMed-MEDLINE, Web of Science, Cochrane, and Scopus were all searched. “3D printing”, “Aligners”, “Artificial intelligence”, “Future trends”, “Orthodontics” and “Teleorthodontics” were the search terms used to access the database. The above keywords represented the majority of articles that were displayed in the search databases when we searched them using the following phrase- “Future trends in orthodontics”.

### 2.7. Data Selection and Coding

Two independent reviewers located the relevant papers by using the right keywords in various databases and online search tools. The chosen articles were compared, and a third reviewer was brought in if there was a dispute.

After choosing the articles, the same two reviewers independently extracted the following data: author, year of publication, country, kind of publication, study topic, population demographics (n, age), outcome measure(s), relevant result(s), and conclusion (s). The data were compared using the SPSS software (version 26.0, Chicago USA) and any differences were discussed with the third reviewer.

After the selection of the studies, case processing summary of selected studies for interrater reliability and the Chi^2^ test for interrater reliability of the selected studies was performed.

### 2.8. Statistical Analysis

After selecting data on the sample size, variables analyzed, and various elements of the investigations, the data were then entered into the Revman 5 program (version 5, Intel, Santa Clara, CA, USA, 2019) for meta-analysis. Forest plots illustrating the odds ratio for different study methodologies were obtained as part of the meta-analysis for our study.

### 2.9. Risk of Bias Assessment

The risk of bias in the papers we picked was assessed using the AMSTAR-2 method [19]. AMSTAR 2 has been made available as a critical evaluation tool for systematic reviews, joining a number of other instruments that have served the same purpose. It consists of a 16-point checklist, as shown in Table 1 below. The domains listed in the Cochrane risk of bias instruments for systematic reviews are identified by the AMSTAR 2 risk of bias items. These show that an agreement was reached in each case following input from more than 30 methodology experts. This tool was employed to assess the effectiveness of our selected studies, since AMSTAR-2 is applicable for reviews consisting of both randomized and non-randomized studies which is applicable for a systematic review such as ours whose major objective is the analysis of trends/treatment modalities in orthodontics that might become mainstream in the coming times, which ultimately means the studies in our review would be primarily of a speculative nature.

## 3. Results

**Figure 2:**
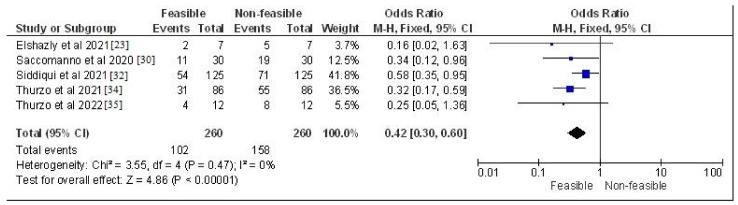
Odds ratio of in-vitro, observational and cohort-based studies selected in this systematic review which assessed the feasibility vs. non-feasibility rates of their respective trends represented on a forest plot after meta-analysis [23,30,32,34,35].

**Figure 3:**
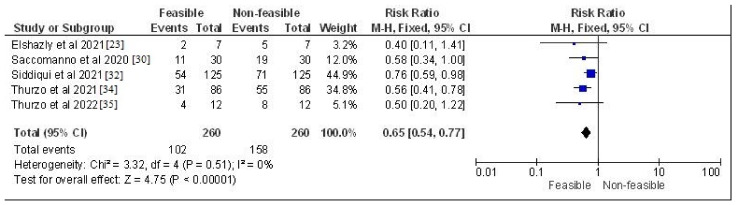
Risk ratio of in-vitro, observational and cohort-based studies selected in this systematic review which assessed the feasibility vs. non-feasibility rates of their respective trends represented on a forest plot after meta-analysis [23,30,32,34,35].

**Figure 4:**
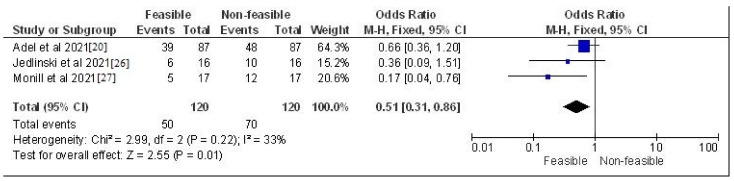
Odds ratio of systematic reviews which assessed the feasibility vs. non-feasibility rates of their respective trends represented on a forest plot after meta-analysis [20,26,27].

**Figure 5:**
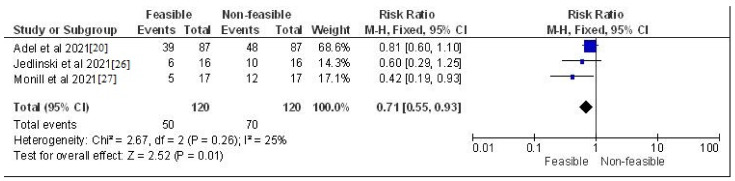
Risk ratio of systematic reviews which assessed the feasibility vs. non-feasibility rates of their respective trends represented on a forest plot after meta-analysis [20,26,27].

The study design, methodology employed, description and outcome are mentioned in Table 2. The results of the meta-analysis are provided in Figure 2, Figure 3, Figure 4 and Figure 5. Moreover, the Chi^2^ test for interrater reliability of the selected studies was performed as mentioned in Table 3 and Table 4 respectively.

## 4. Discussion

Observing orthodontic literature, it is anticipated that 3D applications will overtake 2D applications as the most often measured domain since clinical applications of scanning, printing, and related software have captured the interest and imagination of both care providers and seekers in this decade [26]. According to the sub-categorization of domains, the 3D applications under our study have paid the most attention to the study of morphological and surface properties. On the other hand, little to no attention has been paid to the use of health resources, biocompatibility (such as the premature polymerization effect of 3D printed materials and the dynamics of ultrafine aerosol emission), occupational hazards, cost-benefit analyses, and patient-reported measures of outcome and treatment safety [28]. The authors do think that in the near future, more space will be devoted to these research fields in published literature [34].

It is fascinating to see that 27% of reported outcomes are influenced by social media and orthodontic marketing. Numerous studies have looked at how patients perceive, are aware of, and use health resources in the context of social media impacts. We sought to address a variety of issues, such as the study of tweets pertaining to orthodontics, the effect of social media on knowledge, patient use of social media, and the influence of media advertising on consumer perceptions [32]. However, there is little to no orthodontic literature that addresses issues such as the veracity of social media, the veracity of educational information, and creative marketing in the era of social media dominance. Without a doubt, it must be acknowledged that this media will have a considerable impact on how orthodontic treatment will be provided in the future. With 20% of the significant consideration, biomaterials, nanotechnology, biometrics, and battery-powered devices came in second. The main applications of nanotechnology have been in orthodontic adhesive fillers composed of nano-composites and nano-ionomers. On the other hand, gecko-inspired brackets, smart brackets, and polymers inspired by mussels garnered less attention and were mostly in the development or proof of concept stages. In influential articles, the first wire-mediated, true-scale Smart bracket was created and mechanically characterized. The idea of using sensor systems to control the 3D-force-moment of orthodontic brackets was quite exciting, but it has not yet been put to the test of telemetric energy and data transfer [37]. A study of similar methodology, but albeit a different objective, was carried out by Venezia et al. [38], where the clinicians aimed to evaluate the accuracy of orthodontic models for the production of clear aligners generated with four 3D printers featuring different technologies and belonging to different market segments. They observed that the accuracy of orthodontic models generated for clear aligners can be influenced by different technologies/market segments of the 3D printers used.

Information on patient education, orthodontic training, and tele-orthodontics was the least well-represented. Teleorthodontics is the use of telecommunications and information technology to facilitate public awareness campaigns and provide patients with particular orthodontic information [39,40]. Hansa et al. carried out a study to assess the use and range of tele-orthodontics [41]. The goal was to examine the impact of appointment efficiency, patient viewpoints, and patient demographics on the use of the remote monitoring software (Dental Monitoring, DM). The DM’s integrated platforms, including a patient-specific mobile app, a patented movement monitoring algorithm, and a web-based doctor dashboard, help to assess the treatment’s progress or post-treatment stability. However, the COVID-19 pandemic, which severely restricted patients’ ability to travel to their clinicians’ offices for treatment because of the extensive lockdown imposed across countries throughout the world in order to contain the virus, has increased the importance of the field of teleorthodontics [30,33,35].

Future orthodontic residents may receive valuable orthodontic teaching through virtual reality (VR) and augmented reality (AR) [42,43]. Although AR and VR in orthodontics are still in their infancy, advancements have been made in other dental specialties [41]. Haptic devices that considerably improve skills in tooth preparation by letting the operator feel the force during treatment. Orthodontic residents could employ AR and VR technologies to carry out virtual tasks such as bonding, inserting mini-implants, and bending wires in environments that closely resemble real life.

Another area where the profession of orthodontics has advanced significantly is using CAD/CAM technologies. In a study demonstrating the effectiveness of this method, Giudice et al. evaluated the fitting of prototyped splints that were digitally created (CAD) with various offset values and produced using two various biocompatible resins [44]. They discovered that the results were similar with both types of biocompatible resins utilized and that the splints with an offset value of 0.20 mm had reduced gap volume and deviation analysis values than those with offset values of 0.15 and 0.25 mm. Ye et al. conducted research on comparable parlance with the main goal of evaluating the accuracy of 3D-printed splints made from various dental model offsets [45]. Similar to Giudice et al.’s study [44], they found that 3D-printed splints made from offset dental models (offset 0.05 mm, 0.1 mm, and 0.2 mm) fitted teeth better than splints made from no-offset dental models.

The lack of randomized control trials can be attributed to be a major flaw in this systematic review of ours. However, the topic of emerging trends in the field of orthodontics as we mentioned are all about technologies that have the potential to become mainstream in the coming years, and that as such limits organizations/researchers from investing in these trends whose success is uncertain. In addition, orthodontic literature is suggestive of the fact that the aspect of changes in communication in orthodontics is quite under-researched, especially with regards to teleorthodontics or social media communication. Hence, we believe that more studies are needed to ascertain the pros/cons of these emerging trends so as to establish their credibility as trends that are beneficial to not just orthodontists but the patients as well.

## 5. Conclusions

It was clear through this systematic review and subsequent meta-analysis of the selected studies that orthodontics as a field is also evolving in sync with the advancement of technology, with techniques such as 3D printing, Teleorthodontics and biopolymers spearheading the changes brought upon the orthodontic landscape. However, all the changes that are happening cannot be gobbled up at once, caution and restraint being the most important aspects that need to be followed while adopting these advancements. Further studies are warranted, especially in cases of social media communication where both the patient and the orthodontic practitioner need to be careful in order to maintain their privacy in an increasingly online world.

## Figures and Tables

**Table 1:** AMSTAR-2 16-point checklist of risk of bias assessment in studies selected for the systematic review.

Studies Selected	Question and Inclusion	Protocol	Study Design	Comprehensive Search	Study Selection	Data extraction	Excluded Studies Justification	Included Study Details	Risk of Bias	Funding Sources	Statistical Methods	Risk of Bias in Meta-Analysis	Risk of Bias in Individual Studies	Explanation of Heterogeneity	Publication Bias	Conflict of Interest
Adel et al. 2021 [20]	Yes	Yes	Yes	Yes	Yes	No	No	No	Yes	N/A	Yes	Yes	Yes	Yes	Yes	Yes
Allareddy et al. 2019 [21]	Yes	Yes	Yes	Yes	Yes	No	No	No	Yes	N/A	Yes	Yes	Yes	Yes	Yes	Yes
Cunha et al. 2021 [22]	Yes	Yes	Yes	Yes	Yes	No	No	No	Yes	N/A	Yes	N/A	Yes	Yes	Yes	Yes
Elshazly et al. 2021 [23]	Yes	Yes	Yes	Yes	Yes	No	No	No	Yes	N/A	Yes	Yes	Yes	Yes	Yes	Yes
Gandedkar et al. 2019 [24]	Yes	Yes	Yes	Yes	Yes	No	No	No	Yes	Yes	Yes	Yes	Yes	Yes	Yes	Yes
Hassan et al. 2021 [25]	Yes	Yes	Yes	Yes	Yes	No	No	No	Yes	Yes	Yes	Yes	Yes	Yes	Yes	Yes
Jedlinski et al. 2021 [26]	Yes	Yes	Yes	Yes	Yes	No	No	No	Yes	N/A	Yes	Yes	Yes	Yes	Yes	Yes
Monill et al. 2021 [27]	Yes	Yes	Yes	Yes	Yes	No	No	No	Yes	N/A	Yes	Yes	Yes	Yes	Yes	Yes
Panayi et al. 2021 [28]	Yes	Yes	Yes	Yes	Yes	No	No	No	Yes	N/A	Yes	N/A	Yes	Yes	Yes	Yes
Park et al. 2021 [29]	Yes	Yes	Yes	Yes	Yes	No	No	No	Yes	N/A	Yes		Yes	Yes	Yes	Yes
Saccomanno et al. 2020 [30]	Yes	Yes	Yes	Yes	Yes	No	No	No	Yes	N/A	Yes	Yes	Yes	Yes	Yes	Yes
Safari et al. 2018 [31]	Yes	Yes	Yes	Yes	Yes	No	No	No	Yes	N/A	Yes	Yes	Yes	Yes	Yes	Yes
Siddiqui et al. 2021 [32]	Yes	Yes	Yes	Yes	Yes	No	No	No	Yes	N/A	Yes	N/A	Yes	Yes	Yes	Yes
Sycinska et al. 2021 [33]	Yes	Yes	Yes	Yes	Yes	No	No	No	Yes	N/A	Yes	Yes	Yes	Yes	Yes	Yes
Thurzo et al. 2021 [34]	Yes	Yes	Yes	Yes	Yes	No	No	No	Yes	Yes	Yes	Yes	Yes	Yes	Yes	Yes
Thurzo et al. 2022 [35]	Yes	Yes	Yes	Yes	Yes	No	No	No	Yes	N/A	Yes	Yes	Yes	Yes	Yes	Yes
Zakrzewski et al. 2021 [36]	Yes	Yes	Yes	Yes	Yes	No	No	No	Yes	Yes	Yes	Yes	Yes	Yes	Yes	Yes

**Table 2:** Description and outcomes as observed in the studies selected for the systematic review.

Author and Year of Study	Sample Size	Study Design	Study Description/Characteristics	Study Outcome/Inference
Adel et al. 2021 [20]	87 studies	Systematic review	Robotic dental assistants, robotics in diagnosis and simulation of orthodontic problems, robotics in orthodontic patient education, teaching, and training, wire bending and customized appliances, nanorobots/microrobots for accelerating tooth movement and for remote monitoring, robotics in maxillofacial surgeries and implant placement and automated aligner production robotics were all examined in this review of published orthodontic literature.	According to the review, there had been significant research in the recent ten years on arch wire bending and tailored appliance robots, simulative robots for diagnosis, and surgical robots (32%, 22%, and 16%). The orthodontic literature had extensively reported on nanorobots and rehabilitation robots, which were both highly promising (13%, 9%). However, additional scientific information will need to be acquired in the future about patient robots, automated aligner manufacturing robots, and assistive robots (1%, 1%, and 6%).
Allareddy et al. 2019 [21]	-	Literature review	This report’s objectives were to present various machine learning techniques, give a summary of the big data analytics market in the healthcare industry, and talk about potential consequences for the orthodontics industry. The traditional analytical methods may no longer be useful for analyzing clinical outcomes due to the growing availability of data from numerous sources.	The current state of big data analytics in the healthcare industry, as well as the typical analytical methods used to analyze massive data sets, were found to be extremely advantageous, the authors concluded. There were various ways that big data analytics could be utilized in orthodontics to enhance clinical outcomes.
Cunha et al. 2021 [22]	-	Literature review	This article’s objective was to provide a description of the resources and clinical uses of CAD/CAM technology in orthodontics.	The use of virtual bracket removal and digital indirect bonding may have shortened the length of orthodontic treatment, eliminated clinical and laboratory stages, enhanced patient comfort, and improved accuracy and predictability. However, the use of CAD/CAM technology in orthodontics came at a higher cost and required specialized training.
Elshazly et al. 2021 [23]	7 aligners	In-vitro study	In this in vitro work, the use of shape memory polymers (SMPs) as the materials for aligners was studied as a novel strategy to get around the rate-limiting staging of traditional aligners. The goal of the study’s design was to move an upper central incisor 1.9 mm in the right direction with just one aligner after several steps/activations. A moving upper central incisor aligned typodont model that was specifically designed for scanning was used. Resin models were produced using orthodontic software and a 3D printer.	The results revealed that the SMPs’ aligner’s overall correction efficiency was around 93%. (1.76 mm). Following the reforming stage, the corrective movement was 0.94 0.04 mm, followed by 0.66 0.07 mm and 0.15 0.10 mm after the first and second activation steps, respectively. It was determined that the use of SMP-based aligners for orthodontic aesthetic treatment has a bright future.
Gandedkar et al. 2019 [24]	-	Literature review	Recent advancements in orthodontic 3D applications, such as 3D printing, diagnosis, and management, recent advancements in orthodontic biomaterials, nanotechnology, biomimetics, battery-driven devices, recent advancements in orthodontic patient education, orthodontic training, and orthodontics practice management, and recent advancements in orthodontics were all examined in this scoping review of published orthodontic literature for the past 10 years (2009–2019). A total of 1245 records were looked up, and 65 potentially pertinent items were fully located. Following screening, 42 studies were included in the scoping review because they satisfied the selection criteria.	The review discovered that studies relating to morphological features or surface characteristics with regard to 3D applications (49% representation)—3D printing, diagnosis, and management—were the most common. The past ten years have seen significant reports on biomaterials, nanotechnology, biomimetics, and battery-driven devices, as well as orthodontic marketing and the influence of social media (27%) and biomaterials. According to the authors, more scientific information was required in the fields of patient education, e-health, tele-orthodontics, and patient confidentiality protection.
Hassan et al. 2021 [25]	-	Literature review	This study intended to analyze recent advancements in BPA-free monomers used in the production of adhesives and resin dental composites. Due to their relevance to potential orthodontic applications, the most promising polymeric smart materials were also highlighted.	According to the authors, recent advancements in polymeric orthodontic materials had the ability to address the shortcomings of earlier materials through increased mechanical characteristics and, most crucially, BPA-free constructions. Additionally, the effectiveness and durability of orthodontic treatments would have been enhanced by the newly discovered family of polymers with intriguing qualities, such as the dual functions of shape-memory polymers, self-healing, self-cleaning, and biomimetic adhesion.
Jedlinski et al. 2021 [26]	16 studies	Systematic review	The goal of this study was to thoroughly examine and synthesize the available controlled studies that looked into the precision and effectiveness of intraoral scanners for orthodontic purposes and that gave clinically valuable information and guided subsequent research in this area. MedLine (PubMed), Scopus, Web of Science, and Embase were used to conduct a literature search utilizing free text and MeSH phrases. Studies on the use of intraoral scanners in orthodontics were found using search engines (from 1950 to 30 September 2020). 16 of the 67 full-text articles that had been assessed for inclusion criteria after duplicates had been removed were ultimately chosen and included in the qualitative synthesis.	There was a great deal of information accessible about the usefulness and accuracy of various scanners. The accuracy of scanners from various manufacturers that belonged to the same generation was nearly identical. Due to this, comparable study would not significantly advance orthodontics in the future. The authors stated that finding further uses for digital impressions in orthodontic treatment will be a challenge in the upcoming years.
Monill et al. 2021 [27]	17 studies	Systematic review	The Preferred Reporting Items for Systematic Reviews and Meta-Analyses Extension for Scoping Reviews (PRISMA-ScR) criteria were followed for conducting this review. The MEDLINE/PubMed, Scopus, Web of Science, Cochrane, and IEEE Xplore databases were used for the electronic literature search, which had an 11-year time limit from January 2010 to March 2021. There were no extra manual searches carried out. The initial 311 records from the electronic literature search were reduced to 115 when duplicate references were removed. Finally, the qualitative synthesis review included 17 papers that qualified when the inclusion criteria were applied.	The studies that were analyzed showed that anatomical reference points on radiological images could be automatically detected using convolution neural networks. The Cervical Vertebral Maturation stage could be identified using a model of an artificial neural network in the field of growth and development, and the results were identical to those of skilled human observers. Additionally, AI technology may enhance the diagnostic efficacy of orthodontic treatments, aiding the orthodontist in performing their work more precisely and effectively.
Panayi et al. 2021 [28]	-	In-vitro study	A 25-year-old healthy male’s complete records, including a 3D intraoral scan, were obtained. The UBrackets^®^ program imported the scan, and digital setup was completed. DTC^®^ virtual lingual brackets were continuously and automatically placed (Hangzhou DTC, China). The brackets were placed in the ideal location using a variety of manipulators (mesiodistal, labiolingual, rotating). The bases of the brackets were almost extruded toward the surface of the teeth. The extrusion reflected the precise quantity of composite that would be affixed to the bases of the brackets to produce the unique brackets. The IDB tray, the archwire, and the models were finally exported. To bend each next archwire, the exporting wire served as a prototype.	The customized bases were made by inserting DTC lingual brackets into the IDB tray and then covering the bracket bases with composite. A custom-bent, 0.012” NiTi archwire was introduced while accurate bonding was being carried out. For the first time, an orthodontist was able to build a virtual bracket base (labial or lingual) in-house using CAD software called Deltaface Ubrackets^®^ (Coruo, Limoges, France), and then transfer the personalized brackets to the patient using a 3D printed indirect bonding (IDB) tray.
Park et al. 2021 [29]	-	Literature review	This article explored several teledentistry system types for orthodontic practices, implementation tips, and significant regulatory considerations regarding the use of teledentistry for orthodontic applications. Prior to committing to a service, a thorough assessment of the software’s intended use was required, the researchers stated. Additionally, the popularity of teledentistry in orthodontics as a way to confer with and monitor a patient without an in-office visit was accelerated by technological improvements, rising patient demand, and the requirement for social isolation due to Coronavirus Disease 2019.	It was necessary to ensure that the assigned clinic computer met the system’s criteria and install all security measures. Teledentistry patients must be located within the clinician’s statutory license boundary, and appointments must be recorded in the same way as in-office visits. Teledentistry required to be mentioned on informed consent forms. Additionally, it was mentioned that while malpractice insurance covered everything typical and customary allowed by the provider’s license, teledentistry enhanced the requirement for cyber liability insurance.
Saccomanno et al. 2020 [30]	30 patients (16 females)	Observational study	The study included 30 individuals who had received various orthodontic treatments conventionally and who the physician was still monitoring via tele-orthodontics. A comparison with patients who underwent no follow-up or solely in-office follow-ups was not possible due to the clear limitations of tele-practice. Videocalls, specialized applications, intraoral and extraoral pictures taken by the patients, instant messaging, and dedicated programs were the communication methods employed in this study and suggested in their concept of tele-orthodontics.	Tele-orthodontics made it possible to complete some orthodontic follow-up procedures with less chairside time, up to a 45-min reduction in patient waiting time, a lower risk of infection, fewer or no missed appointments, targeted problem-solving, and more follow-ups with patients who are odontophobic. Overall, the benefits of tele-orthodontics outweighed the drawbacks of in-person visits and fewer personal interactions.
Safari et al. 2018 [31]	-	Literature review	This study analyzed the most recent and potential uses of stem cells (SCs) in orthodontics and dentofacial orthopaedics because both fields are related to dentofacial orthopaedics, which involves bone regeneration.	It was found that SCs might be applied to repair infrabony alveolar defects and relocate teeth into the restored regions. SCs will likely be used in orthodontics in the future to broaden movement restrictions, regenerate resorbed roots, and speed up tooth movement. However, this assessment found that the evidence for these roles was insufficient, and more research was needed to assess if these theories would be viable.
Siddiqui et al. 2021 [32]	125 patients (83 females)	Prospective cross-sectional study	In this investigation, each participant answered questions about their knowledge of, access to, and use of social media, as well as their readiness to use it to promote orthodontic treatment. There were neither eligibility requirements nor age limits.	The patients had access to social media in 99% of cases. 30% of these patients had used social media in relation to orthodontic therapy, with Instagram (n = 17) and Snapchat (n = 12) being the most common platforms. Of these patients, 64% were aware that social media platforms were accessible to aid in orthodontic treatment. 73% of the patients said they would be open to using social media to promote orthodontic treatment in the future. Social media was found to be interesting, approachable, and adaptable, and it had been shown successful at increasing patients’ knowledge about orthodontic treatment.
Sycinska et al. 2021 [33]	-	Scientometric analysis	This study looked into how orthodontic treatment was affected by the COVID-19 pandemic epidemic. Using Google Trends, the data regarding orthodontic queries was examined in real-time. Search phrases related to the year before the pandemic outbreak and the time of the epidemic were examined. As another example of various orthodontic appliances, the five-year trend for queries “braces” vs. “invisalign” was contrasted.	Due to the many announcements of limits and lockdowns in the spring of 2020, there was a considerable drop in orthodontics keyword searches. During the initial lockdown in 2020, there was less interest in the question “braces pain.” While the number of searches for “braces” remained largely consistent across the analyzed time period, the number of searches for “invisalign” increased considerably over time. It was determined that the COVID-19 pandemic’s progression significantly influenced the search queries for orthodontic-related terms.
Thurzo et al. 2021 [34]	86 subjects (54 females)	Observational study	This study set out to assess the clinical effects of an AI enhancement to existing orthodontic mobile coaching software. The secondary objective was to describe the benefits of telemonitoring systems for clinical effect evaluation and the deployment of AI (decision tree process algorithm).	Except for the male manifestation of clinical non-tracking as determined by artificial intelligence from video scans, all variables significantly improved after the update. According to the authors, updating existing health care applications to include computerized decision processes greatly improved clinical performance and patient compliance. It was discovered that the approach may have established a baseline for further machine learning optimization.
Thurzo et al. 2022 [35]	12 completed treatments	Observational study	The purpose of this study was to compare two different biocompatible photopolymers and evaluate the practicality of using 3D printed distalizers in clinical settings (white and transparent). On the set of 12 full orthodontic treatments, the frequency of distalizers debonding and patients’ perceptions of aesthetics were assessed. A bonded distalizer treatment time averaged 6.4 months in length. All of the cases involved adults with unilateral Class II malocclusions that were treated using a hybrid strategy as part of the all-encompassing Invisalign^®^ program.	The outcomes demonstrated the viability of perspective practice for 3D design and in-office 3D printing of a customized distalizer. Additionally, the findings revealed no clinically relevant variations between the two biopolymers under study. The study came to the conclusion that dental resin additive manufacturing was a practical technique for customizing and in-office 3D printing of orthodontic accessories, notably distalizers. New materials for 3D printing in orthodontics enable greater individualization and more effective treatment.
Zakrzewski et al. 2021 [36]	-	Literature review	This study concentrated on the idea of nanotechnology and its applications in the field of orthodontics, such as, for example, improving the antibacterial properties of orthodontic resins, resulting in a decrease in enamel demineralization, or controlling friction force during orthodontic movement.	This study demonstrated the use of nanoparticles in orthodontics for their mechanical and antibacterial capabilities. To successfully stop enamel demineralization during orthodontic therapy, nanoparticles can be introduced to acrylic resins, cements, or orthodontic adhesives. The need of managing orthodontic therapy under control and in concert was also noted by the researchers.

**Table 3:** Case processing summary of selected studies for interrater reliability.

Case Processing Summary						
	Cases					
	Valid		Missing		Total	
	N	Percent	N	Percent	N	Percent
Cases included in the systematic review	17	100.0%	0	0.0%	17	100.0%

**Table 4:** Chi^2^ test for interrater reliability of the selected studies.

	Value	df	Asymptotic Significance (2-Sided)
Pearson Chi-Square	204.000 ^a^	192	0.263
Likelihood Ratio	85.239	192	1
N of Valid Cases	17		

where ^a^ 221 cells (100.0%) have expected countless than 5. The minimum expected count is 0.06.

## Data Availability

All data are available within the manuscript.
